# Goal Orientation and Physical Activity: Psychometric Properties of the Polish Version of the Goal Orientation in Exercise Measure (GOEM)

**DOI:** 10.3390/jcm10091900

**Published:** 2021-04-28

**Authors:** Maciej Tomczak, Paweł Kleka, Aleksandra Walczak, Łukasz Bojkowski, Małgorzata Walczak

**Affiliations:** 1 Department of Psychology, Poznan University of Physical Education, Królowej Jadwigi 27/39, 61-871 Poznań, Poland; bojkowski@awf.poznan.pl (Ł.B.); walczak@awf.poznan.pl (M.W.); 2 Faculty of Psychology and Cognitive Sciences, Adam Mickiewicz University in Poznan, Szamarzewskiego 89, 60-568 Poznań, Poland; pawel.kleka@amu.edu.pl; 3 Heliodor Święcicki Clinical Hospital of the Poznań University of Medical Sciences, Przybyszewskiego 49, 60-355 Poznań, Poland; aleksandra.i.walczak@gmail.com

**Keywords:** physical activity, goal orientation, validity, GOEM

## Abstract

Motivational factors are among the most important determinants of undertaking and participating in physical activity. An adequate measurement of motivation and the assessment of its initial characteristics form the basis for possible further practical interventions of a compensatory or promotional nature. Considering the above, the main aim of this study was to assess the psychometric properties of the Polish version of the Goal Orientation in Exercise Measure (GOEM), and to determine the association between the task and the ego orientations and selected components of physical activity, i.e., frequency of undertaking, declared time per session and time spent on physical activity. In addition to the typical indices of psychometric assessment, an analysis of the reliability of test items by applying the item response theory (IRT) model was also presented. The study included 318 individuals (173 females, 145 males), aged 21 years on average, undertaking recreational physical activity. The GOEM scale by Petherick and Markland (2008) was used. The model of the Polish version of the GOEM scale fitted the empirical data well (CFI = 0.955). Satisfactory reliability indices were also obtained (task subscale: alpha = 0.83, omega = 0.83; ego subscale: alpha = 0.86, omega = 0.86). Reliability, as assessed by the test-retest method, was ICC = 0.80 for the task subscale and ICC = 0.87 for the ego subscale. The IRT analysis showed that the ego subscale was more precise in the middle level of the trait, while the task subscale provided more information in the low and middle level of the trait and relatively little information in the high level of the trait. The positive associations of the task subscale with the frequency of physical activity and the time spent in a single exercise session were also noted.

## 1. Introduction

Regular physical activity is an important prerequisite for health maintenance and disease prevention. Active people are generally not only more functionally fit and less likely to experience health problems, but also feel better and ultimately have a higher sense of the quality of life compared to less active people. Hence, issues concerning the determinants of physical activity and its long-term continuation, as well as its course, seem to be extremely important for people of different ages [1,2,3,4].

Among the determinants of participation and the course of physical activity, motivational factors are very important, and among them, components of autonomous motivation in particular [5]. The importance of goal orientation is also highlighted [6,7]. It derives from Nicholls’ [8] theory, developed in educational and sporting contexts, which assumes the presence of task and ego orientations. In sport, where one of the most important features is competition, this approach has been adapted and, moreover, accurate and reliable measurement methods have been constructed. These include the Task and Ego Orientation in Sport Questionnaire (TEOSQ) [9,10] and the Perception of Success Questionnaire (POSQ) in particular [11,12].

Embedding Nicholls’ [8] approach in the context of physical activity [13], it can be concluded that people with a task orientation experience more pleasure and satisfaction when they feel their skills progress during exercise. They also experience satisfaction when they have a sense of achievement from an exercise-related goal. They enjoy the state of intense involvement in an exercise, trying to perform as well as possible. In contrast, individuals who are characterized by an ego orientation during exercise feel justified when they perform the exercise better than other participants, and can manifest superiority in this regard [8,9,13,14,15]. Participation in physical activity seems to be naturally related to task orientation [16,17]. It can be assumed that an orientation towards personal development, progression and the achievement of goals related to physical tasks promotes involvement in physical activity. These components belong to the task subscale, which is positively correlated with the intrinsic motivation [13,17,18], which, in turn, is important for physical activity functioning [19]. Additionally, according to Kilpatrick [16], the associations of ego orientation with the exercise context can be explained by referring, for example, to social comparison theory [20]. This assumes, among other things, that people compare themselves with other people in different areas of their lives; however, the context for engaging in physical activity is strictly psychosocial in nature. For people with a high level of ego orientation, comparing themselves with people who are less competent in exercise may enhance their self-esteem. In turn, people who relate their skills to more competent individuals may experience an effect of increasing motivation to exercise [16]. This aspect of interpersonal comparisons during exercise may be facilitated by easy opportunities to assess the degree to which each participant in physical activity is performing a given exercise. Thus, the activity of individuals with high ego orientation can be easily regulated by identifying the level of task performance of other exercisers in their area.

The search for possibilities to measure goal orientation in the area of physical activity led Kilpatrick [16] to develop his own Goal Orientation in Exercise Scale (GOES). The scale was constructed using the Task and Ego Orientation in Sport Questionnaire (TEOSQ) [9]. Wording changes were made in some items to emphasize the exercise context. New items were also created. It was indicated that the final two-factor model (5 ego and 5 task items) fitted the empirical data well in the confirmatory factor analysis. The internal consistency of subscales was also satisfactory. It was also shown that the task subscale was slightly positively associated with the duration of exercise, exercise intensity, and enjoyment of exercise. Additionally, women appeared to have lower scores on the ego subscale than men. Higher scores on the ego subscale were typical for those who declared that their motive for exercising was competition [16].

In order to improve the measurement of goal orientation in the area of physical activity, Petherick and Markland [13] created the GOEM scale. The justification for the need to create a new scale can be found in the authors’ work [13]. In the new scale, the items of the task subscale concerned the feeling of competence related to one’s own performance and personal improvement. On the other hand, the items of the ego subscale concerned the feeling of higher competence during exercise compared to other participants. First, 21 test items (9 for the task subscale, 12 for the ego subscale) were selected from the GOES scale and the TEOSQ questionnaire, and then assessed by four experts who were well acquainted with achievement goal theory and the psychology of physical activity. Moreover, in the scale instructions, the emphasis was put on the circumstances in which the respondent is feeling that everything is going well while participating in physical activity. The study included 372 recreational physical activity participants (248 females and 124 males). Initially, after the confirmatory factor analysis, 11 test items were eliminated based on residual analysis and modification indices. The model for the adjusted scale containing 10 items (5 for the task subscale, 5 for the ego subscale) obtained a very good fit to the data and satisfactory Cronbach’s alpha coefficient values. Moreover, it was shown that the model was invariant across gender. It was also indicated that men had higher scores on the ego subscale than women [13]. The correlations between the task and the ego subscale scores, measured by the GOEM scale, and the Behavioral Regulation in Exercise Questionnaire-2 (BREQ-2) [21] subscales scores were also tested. The positive correlations of the task scores, with both intrinsic (e.g., “I exercise because it’s fun”) and identified regulation (e.g., “I value the benefits of exercise”) scores, were found, as well as a positive but lower correlation of the task scores and the introjected regulation (e.g., “I feel guilty when I don’t exercise”) scores. Negative correlations were found between the task scores with amotivation (e.g., “I don’t see why I should have to exercise”) as well as external regulation (e.g., “I exercise because others say I should”) scores. Positive, small correlations of the ego subscale scores with external and introjected regulation were also found [13]. These results provide support for the validity of the GOEM scale.

The validity and reliability of the GOEM scale were also confirmed in the Portuguese [17] and Turkish studies [18]. For example, the CFA models of these versions of the GOEM scale fitted the data very well [17,18]. In the case of the Portuguese version of the scale, the authors obtained the results indicating a good fit of the model to the data for the whole group, women and men separately, fitness group classes, cardio workout and resistance training. The results of invariance analysis in the specified groups were also presented [17]. Furthermore, satisfactory test-retest reliability indices were obtained (for the Turkish version of the scale at a two-week interval, and for the Portuguese version at a four-week interval). The goal orientation scores of the scale tested in the Turkey and Portugal cultural contexts correlated with the different motivation components measured by the Behavioral Regulation in Exercise Questionnaire-2 (BREQ-2) [17,18].

Taking into account the need for goal orientation assessment in the physical activity area in Poland, the main aim of this study was to present the validity and reliability indices of the Polish version of the GOEM scale and to determine the relationships between task and ego scores and the declared frequency of undertaking physical activity, experience in undertaking physical activity (number of months of training) and the duration of one training session.

## 2. Material and Methods

### 2.1. Study Subjects

The study included 318 individuals (173 females, 145 males) with an average age of 21.05 (SD = 2.21), undertaking recreational physical activity. The individuals covered by the study declared that they have undertaken physical activity e.g., for health, appearance, etc. on average 4.12 times per week. The average duration of a training session was 77.7 min, while the average length of time since they began regularly exercising was 86.5 months. They participated in the following disciplines: running, football, gym workout, swimming, dancing, cycling, aerobics, fitness, volleyball, yoga and basketball. The questionnaires were filled out by young people who were firstly verified as participants in different forms of physical activity, both in the academic area and in other areas of physical activity (e.g., fitness centers or other organized groups). The samples were gathered via the internet, mostly during live online meetings which enabled eventual support from researchers. Information about the ongoing study and an invitation to participate was passed along by instructors and coaches associated with the Poznań University of Physical Education.

### 2.2. Research Methods

The Goal Orientation in Exercise Measure (GOEM) questionnaire by Petherick and Markland [13] was used. The scale is used for ego and task goal orientation measurement in the area of physical activity. It consists of 10 statements, where 5 relate to the ego orientation and 5 to the task orientation. Each statement is accompanied by a five-point scale on which the respondent indicates their degree of agreement with the statement. A more detailed description of the scale construction is presented in the introduction to this paper. The research was conducted anonymously on the Internet (online questionnaire). Each respondent received information indicating that the study is voluntary, anonymous, and the results will be used only for scientific purposes. According to the bioethics committee’s opinion, this type of research does not require formal consent. The scale was translated into Polish by an English language expert. Another English expert performed the back-translation of the scale. Then the committee of three sports psychologists and one English translator agreed on the final version of the scale.

Questions on age, gender, and some components of physical activity were included before the main questionnaire items. Respondents were asked how many times a week they undertake physical activity for health, beauty, etc. (not related to professional sports), how long on average one training session takes, and how long they have been exercising (how many years and months).

In order to assess the test reliability, some participants were surveyed twice at a two-week interval. A total of 171 respondents completed the questionnaire for the second time.

### 2.3. Statistical Analysis

Confirmatory factor analysis was used to verify the theoretical validity of the scale. Due to the failure to satisfy the multivariate normality of the distribution, the robust Satorra–Bentler chi-square test was conducted. CFI, TLI and NFI values above 0.95 (or 0.90 based on less restrictive criterion) and RMSEA below 0.08 are assumed to indicate a satisfactory fit of the model to the data [22,23]. Additionally, measurement invariance across gender was tested. First, configural invariance (factor structure consistency) was tested, followed by metric invariance (factor loading consistency), and then scalar invariance (fixed intercept), as well as strict invariance (fixed residual). It was assumed that a decrease in CFI below 0.01 and an increase in RMSEA above 0.015 indicated a lack of measurement equivalence [24].

Cronbach’s alpha and McDonald’s omega coefficients for each subscale were calculated to determine the scale reliability. The reliability was also tested by the test-retest at a two-week interval. The study was conducted on a group of 171 subjects. The intraclass correlation coefficient (ICC) was used to determine correlations between measurements. Additionally, in order to compare the obtained results with those obtained by other researchers, the Pearson correlation coefficient was assessed.

To assess the quality of the test items, the item–rest discriminatory power coefficient and the item response theory model (IRT) (generalized partial credit model) were used. The high value of the information function of the IRT model compared to other values, and the high-hanging line on the graph for a given test item indicate a significant amount of information provided by the item for different levels of trait. Flat and low-hanging lines, and smaller values of the information function indicate lower reliability of the test item (providing less information) [25,26].

The Pearson correlation coefficient was used to assess the correlation between goal orientation and the physical activity components (frequency of undertaking per week, length of one exercise session in minutes, and overall exercise experience in months). In addition to the *p*-value, a 95% confidence interval constructed by using the percentile bootstrap method was presented (5000 samples were used). Multiple regression was used to estimate the association between physical activity components and goal orientation under control of age in gender, in total. It was assumed that the components of physical activity are dependent variables, while ego/task orientation, age and gender are independent variables. The t-test for independent data was used to compare men and women in terms of goal orientation. R package (4.04 version, R Fundation for Statistical Computing, Vienna, Austria), SPSS (27 version, Armonk, IBM Corp., New York, NY, USA) and Statistica (13.3. version, TIBCO Statistica^TM^, Palo Alto, CA, USA) software were used for the analyses.

## 3. Results

### 3.1. Confirmatory Factor Analysis (CFA)

**Figure 1:**
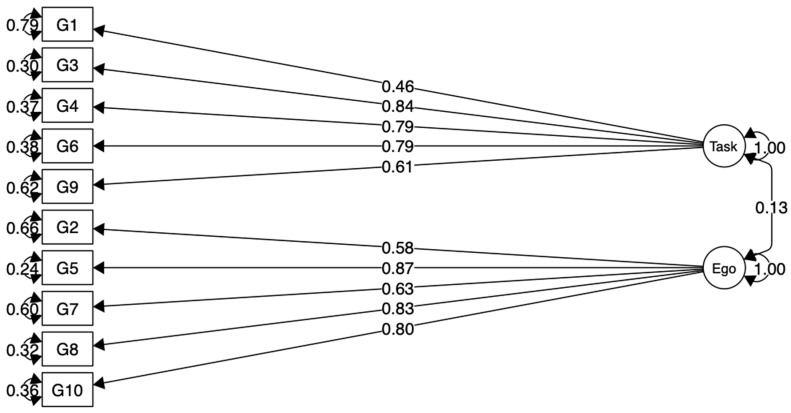
Model CFA for the GOEM scale.

The model of the Polish version of the GOEM scale obtained a good fit for the empirical data (Figure 1, Table 1).

### 3.2. Invariance by Gender

All models obtained a relatively good fit to the data (Table 2). Model 1 obtained a good fit to the data and it indicated a configural invariance across genders. Analysis of the CFI and RMSEA differences between adjacent models indicated that there are no differences between model 1 and model 2. It can be concluded that the metric equivalence of measurement has been achieved. Next, the scalar invariance was tested. Based on the changes in CFI between model 2 and model 3 (decrease greater than 0.01), it can be concluded that scalar equivalence was not obtained, although the changes in RMSEA did not exceed the threshold (0.015). We can conclude that the item intercepts are not similar for people of different genders (Table 3). The intercepts with the highest modification indexes were then released (items 7, 10). There were no highly significant differences between the model with released intercepts (model 4) and the model for metric invariance (model 2) (delta CFI < 0.01). It indicates that partial scalar invariance was obtained. The strict invariance was then tested (additionally fixed residual). No differences between model 4 and model 5 were obtained, which indicate a strict invariance across genders (Table 3). Due to the partial scalar invariance, comparing a latent mean across genders should be treated with caution.

In general, men had higher scores on the ego subscale than women (Women: M = 2.49, sd = 1.03; Men: M = 2.81, sd = 0.95; t(316) = −2.78, *p* < 0.01, Hedges g = 0.32). There were no significant differences in the scores on the task subscale (Women: M = 4.30; sd = 0.62; Men: M = 4.23, sd = 0.62; t(316) = 1.03, *p* > 0.05).

### 3.3. Reliability of the GOEM Scale—Cronbach Alpha, McDonald Omega and Test-Retest Correlation

Both alpha and omega coefficients for the task subscale scores were 0.83 and for the ego subscale scores were 0.86. Item—total correlation for the ego subscale was: 2—0.52, 5—0.80, 7—0.57, 8—0.77, 10—0.72 and for the task subscale was: 1—0.43, 3—0.73, 4—0.69, 6—0.71, 9—0.58.

The correlation between the first and the second measurement was ICC = 0.80 for the task subscale and ICC = 0.87 for the ego subscale (r-Pearson coefficient: task: 0.80, *p* < 0.001; ego: 0.87, *p* < 0.001).

### 3.4. Reliability of Items—IRT Model Used

**Figure 2:**
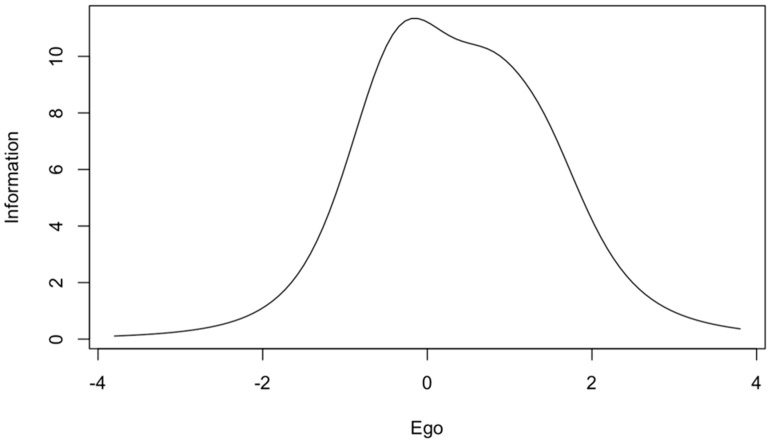
Information curve for the ego subscale of the GOEM scale.

**Figure 3:**
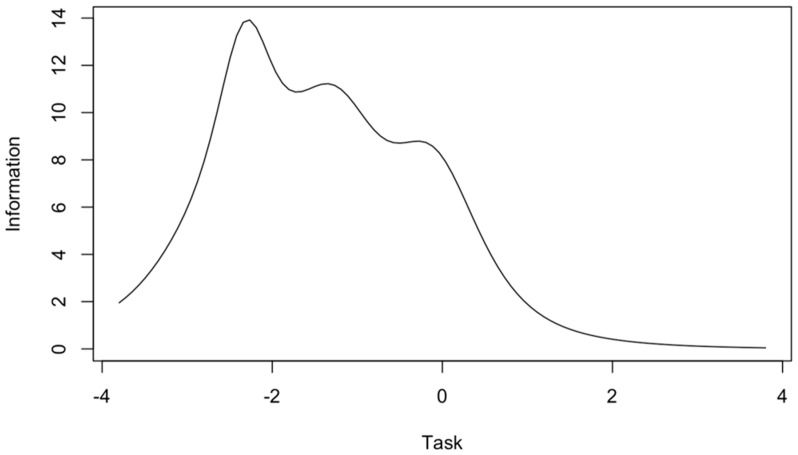
Information curve for the task subscale of the GOEM scale.

**Figure 4:**
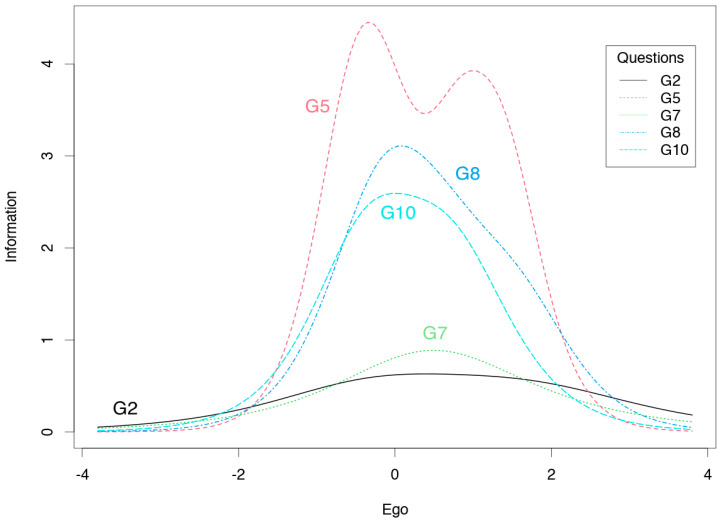
Information curves for the ego scale items of the GOEM scale.

**Figure 5:**
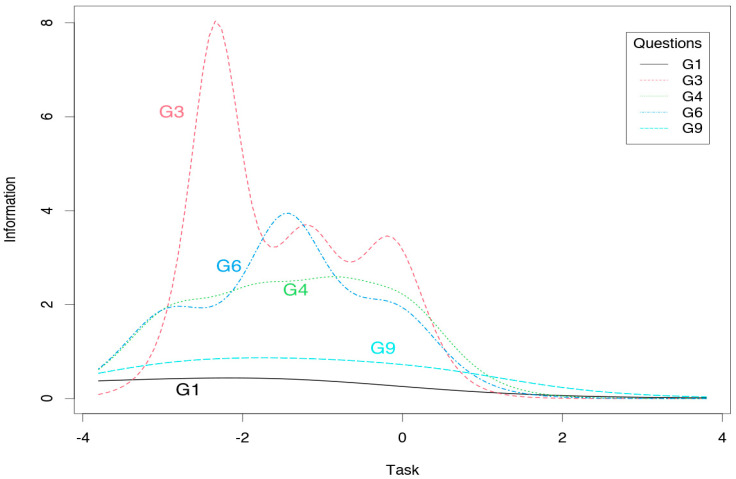
Information curves for the task subscale items of the GOEM scale.

The ego subscale scores provide a great deal of information on the average level of the measured trait, and less information on the low and high levels of each trait (Figure 2). The task subscale results provide little information on the high level of each trait but more information on the low and average level (Figure 3). Smaller values of the information function than the other items on the ego subscale have items 2 and 7 (Table 4), which provide less information in the whole spectrum of the examined trait (Figure 4). For the task subscale, less information is provided by items 1 and 9 (Table 4, Figure 5—flatter curves).

### 3.5. Relations of the Goal Orientation with Physical Activity Components

Statistically significant positive correlations of the task orientation with frequency of physical activity per week, and the length of one training session in minutes, were observed. On the other hand, there were no significant associations between the ego orientation and physical activity components (Table 5). Associations between variables were also demonstrated in regression models, including physical activity components as dependent variables, the ego and the task subscale scores as predictors, as well as the age of subjects and gender as covariates.

The model explains about 3.4 percent of the variance in frequency of undertaking physical activity. A statistically significant positive effect for the task score was obtained (Table 6).

The variables also explain 12.5 percent of the variance in the declared time of one session of physical activity. The positive effect of task orientation and the gender effect for time of one session was also significant (men had higher scores in declared time than women) (Table 7).

The variables also explain about 3.9 percent of the variance in experience in undertaking physical exercise; however, a significant positive effect was observed for age and gender (men had a higher score than women) (Table 8).

## 4. Discussion

Confirmatory factor analysis of the Polish version of the questionnaire provided a satisfactory model fit to the data. In the case of the original version of the scale, as well as the Portuguese and the Turkish versions, the models appeared to provide a better fit to the data [13,17,18]. However, in the context of the assumed indicators, the model obtained for the Polish version of the GOEM scale is acceptable. Moreover, the fit indices for the Polish version of the GOEM questionnaire were better than the fit indices for the Polish versions of the TEOSQ and POSQ questionnaires used to determine goal orientation in sport [14,15]. Furthermore, by analyzing the changes in CFI and RMSEA, the configural, metric and strict equivalence of the gender measurement of the GOEM questionnaire can be assumed. Scalar invariance was only partially demonstrated. Thus, comparisons of latent mean values of the task and the ego subscale in the groups of women and men should be treated with caution. On the other hand, the release of two intercepts (for items 7 and 10) allowed the attainment of partial scalar invariance.

Satisfactory reliability indices were demonstrated. The alpha and omega coefficients were relatively high. The alpha value for the task subscale scores was higher than the value obtained by the authors of the original version of the scale, while the alpha value for the ego subscale was slightly lower than the value obtained by the authors [13]. Compared to the Turkish version, the lower alpha value was obtained for the ego subscale and the task subscale [18]. Furthermore, satisfactory reliability of the Polish version of the scale measured by the test-retest method was obtained, although the values are lower in the case of the task subscale and slightly lower in the case of ego subscale than those obtained for the Turkish version [18].

Additionally, the reliability of the test items was analyzed by applying the IRT. The ego subscale provided a large amount of information for the average level of the measured trait, while the task subscale provided more information for lower and average values of the measured trait. These results are largely consistent with previously obtained IRT results for Polish versions of the TEOSQ and POSQ questionnaires [14,15]. In particular, it is worth considering how the reliability of the task subscale items could be improved in high trait values. Items 2 and 7 in the ego subscale carry less information, likewise in the case of task subscale items 1 and 9. It is currently difficult to compare these results with those of other authors, due to the infrequency of the application of the IRT. However, further research in this area would be worthwhile.

The obtained results show that men had higher ego subscale scores than women; however, no gender differences were observed in the case of the task subscale. This result is consistent with the result obtained by the authors of the original version of the GOEM scale [13]. This effect was shown, among others, by Kilpatrick in his GOES scale research [16]. It was also revealed in research on the Polish validation of the POSQ scale in sport [15]. This aspect might, for example, have an evolutionary as well as a social basis. As interpreted by Petherick and Markland [13], this may be caused by men’s tendency to want to demonstrate their abilities more than women. Furthermore, men are generally more focused on winning than women [13]. Hence, comparisons between men rather than between other exercisers may be more meaningful for them than for women, even when they undertake health-related recreational physical activity. On the other hand, due to the lack of full scalar equivalence of the measurement, comparing a latent mean across genders should be treated with caution.

In both correlation and regression analyses there were also observed positive but small associations between task orientation with the reported frequency of physical activity per week, and the declared time of one training session. These associations seem reasonable, as an orientation towards personal development and improvement of one’s skills may favor more frequent participation in physical activity and longer training sessions. In contrast, there were no associations of goal orientation with the number of months of exercise participation. However, a small positive correlation between the experience of physical activity (years of exercise) and level of task orientation, as determined by the GOES scale, was reported by Kilpatrick et al. [16]. The authors also showed a small positive association of task orientation with exercise intensity and enjoyment of exercise [16]. Associations of goal orientation measured by different scales with different components of physical activity have been repeatedly demonstrated. For example, a review of the literature on the correlates of physical activity in youth also indicated that in general, goal orientation/motivation is related to physical activity [27]. Grasten and Watt [28], on the other hand, showed that the task motivational climate during physical education lessons is positively associated with light and moderate physical activity intensity. Tzetsis et al. [29] also showed that students with higher task orientation (independent from ego orientation level) took longer to engage in vigorous physical activity than did students with low task and high ego orientation. These associations (as presented in the introduction) are quite consistent with previously obtained correlations between autonomous motivation (a positive correlate of task orientation) and various components of physical activity [19].

In general, it can be stated that the Polish version of the GOEM scale has obtained satisfactory psychometric indices in both the aspect of validity and of reliability. Moreover, it was found that task-oriented people generally exercise more often per week, and exercise longer during a single session. However, the study is not free of limitations. Although configural, metric, and strict equivalence was demonstrated, the scalar equivalence of measurement between genders was only partly obtained. Therefore, comparisons of latent mean scores on the subscales of the Polish version of the GOEM scale in women and men should be approached with caution. Additionally, it is worth checking the IRT results on other samples. In the Polish versions of the scales for the measurement of goal orientation in sport (TEOSQ, POSQ) and their time spent in physical activity (GOEM), a lack of differentiation of the subjects in the upper scores of the task subscale has been noted. It is worth establishing whether this issue concerns only the Polish versions of the scales or whether it is a general aspect of the task subscale measurement. In addition, the survey was conducted on young people. In the future, it is also worthwhile to carry out analyses among active middle-aged and older people. On another note, the study presented here was conducted online; thus, the measurement equivalence of the GOEM scale in groups studied via the Internet and by traditional methods is also worth checking.

## Figures and Tables

**Table 1:** The GOEM scale model fitting indices to empirical data.

	ML	Robust Values (S–B)
Chi-sq	99.880	91.699
df	34.000	34.000
*p* value	0.000	0.000
CFI	0.953	0.955
TLI	0.937	0.940
NFI	0.930	0.926
RMSEA	0.078	0.076
RMSEA L.L.	0.061	0.058
RMSEA U.L.	0.096	0.095

S–B—based on Satorra–Bentler chi-square; CFI—Comparative Fit Index; TLI—Tucker-Lewis Index; NFI—Normed Fit Index; RMSEA—Root Mean Square Error Approximation.

**Table 2:** Invariance for the GOEM scale split by gender.

	Chi-Sq Scaled	df Scaled	*p*-Value Scaled	Robust CFI	Robust RMSEA
Model 1. Configural	126.708	68	<0.001	0.954	0.076
Model 2. Metric	136.107	76	<0.001	0.953	0.073
Model 3. Scalar	161.474	84	<0.001	0.941	0.078
Model 4. Scalar part	147.130	82	<0.001	0.950	0.073
Model 5. Strict	156.653	92	<0.001	0.948	0.070

Robust values based on Satorra-Bentler chi-square.

**Table 3:** Differences between models.

	Chisq	Df	ΔCFI *	ΔRMSEA *	ChiSqΔ (df)	*p*-Value
Model 1. Configural	136.470	68	-	-	-	-
Model 2. Metric	147.058	76	0.001	0.003	9.542 (8)	0.2986
Model 3. Scalar	170.791	84	0.012	0.005	28.205 (8)	0.0004
Model 4. Scalar part	157.121	82	0.003	0.000	11.072 (6)	0.0861
Model 5. Strict	173.806	92	0.002	0.003	11.502 (10)	0.3197

Comparison models, deltas: 1–2, 2–3, 2–4, 4–5. * Δ based on robust CFI and RMSEA.

**Table 4:** The value of the information function for individual items.

Ego			Task		
ITEMS	Information (−4, 4)	Proportions of Information	ITEMS	Information (−4, 4)	Proportions of Information
G2	3.03	0.9278	G1	2.03	0.7148
G5	11.99	0.9999	G3	13.87	0.9992
G7	3.13	0.9547	G4	9.76	0.9905
G8	8.68	0.9984	G6	10.02	0.9844
G10	7.10	0.9979	G9	4.47	0.8941

**Table 5:** Correlations between the ego and the task subscale and studied physical activity components.

	Ego (Bootstrap 95% CI)	Task (Bootstrap 95% CI)
Length of one exercise session in minutes	0.08 (−0.0358, 0.1945) *p* = 0.153	0.21, (0.1111, 0.3037) *p* < 0.001
Frequency of undertaking per week	0.08, (−0.0284, 0.1912) *p* = 0.141	0.19, (0.0800, 0.2879) *p* < 0.001
Exercise experience in months	0.06, (−0.0485, 0.1739) *p* = 0.262	0.03, (−0.0943, 0.1387) *p* = 0.646

**Table 6:** Regression model for the frequency of undertaking physical activity (per week).

*n* = 318	R = 0.22, R^2^ = 0.047, Adj. R^2^ = 0.034, F(4.313) = 3.84, *p* < 0.0046				
	β (SE)	B (SE)	t (313)	*p*	Bootstrap 95% CI
Intercept		1.03 (1.05)	0.98	0.3268	−1.3122, 3.3509
Ego	0.05 (0.06)	0.07 (0.08)	0.84	0.3986	−0.0949, 0.2546
Task	0.19 (0.06)	0.46 (0.14)	3.33	0.0010	0.2059, 0.7252
Gender	0.08 (0.06)	0.26 (0.17)	1.49	0.1358	−0.0759, 0.6019
Age	0.06 (0.06)	0.04 (0.04)	1.03	0.3014	−0.0454, 0.1266

**Table 7:** Regression model for the declared time of one session of physical activity (in minutes).

*n* = 318	R = 0.37, R^2^ = 0.136, Adj. R^2^ = 0.125, F(4.313) = 12.33, *p* < 0.0001				
	β (SE)	B (SE)	t (313)	*p*	Bootstrap 95% CI
Intercept		41.84 (18.62)	2.25	0.0254	8.5161, 76.1003
Ego	−0.00 (0.05)	−0.01 (1.51)	−0.01	0.9918	−3.1904, 3.1741
Task	0.22 (0.05)	10.28 (2.43)	4.22	<0.0001	5.7140, 14.7587
Gender	0.29 (0.05)	16.44 (3.06)	5.37	<0.0001	10.5725, 22.2988
Age	−0.06 (0.05)	−0.73 (0.68)	−1.08	0.2829	−2.0396, 0.5589

**Table 8:** Regression model for experience in undertaking physical activity (duration of exercise in months).

*n* = 318	R = 0.22, R^2^ = 0.051, Adj. R^2^ = 0.039, F(4.313) = 4.19, *p* < 0.0026				
	β (SE)	B (SE)	t (313)	*p*	Bootstrap 95% CI
Intercept		−14.34 (37.14)	−0.39	0.6996	−87.2995, 57.1926
Ego	0.04 (0.06)	2.11 (3.02)	0.70	0.4858	−3.7973, 8.0298
Task	0.04 (0.06)	3.21 (4.86)	0.66	0.5085	−6.5072, 12.9181
Gender	0.19 (0.06)	20.34 (6.11)	3.33	0.0010	8.3682, 31.8158
Age	0.14 (0.06)	3.43 (1.36)	2.52	0.0121	0.6527, 6.4410

## Data Availability

The data presented in this study are available on request from the corresponding author.
